# Reconstruction of 3D Object Shape Using Hybrid Modular Neural Network Architecture Trained on 3D Models from *ShapeNetCore* Dataset

**DOI:** 10.3390/s19071553

**Published:** 2019-03-31

**Authors:** Audrius Kulikajevas, Rytis Maskeliūnas, Robertas Damaševičius, Sanjay Misra

**Affiliations:** 1 Department of Multimedia Engineering, Kaunas University of Technology, 51368 Kaunas, Lithuania; audrius.kulikajevas@ktu.edu; 2 Centre of Real Time Computer Systems, Kaunas University of Technology, 51368 Kaunas, Lithuania; rytis.maskeliunas@ktu.lt; 3 Department of Software Engineering, Kaunas University of Technology, 51368 Kaunas, Lithuania; 4 Department of Electrical and Information Engineering, Covenant University, Ota 1023, Nigeria; 5 Department of Computer Engineering, Atilim University, Ankara 06830, Turkey; sanjay.misra@atilim.edu.tr

**Keywords:** 3D depth shape recognition, 3D depth scanning, RGB-D sensors, hybrid neural networks

## Abstract

Depth-based reconstruction of three-dimensional (3D) shape of objects is one of core problems in computer vision with a lot of commercial applications. However, the 3D scanning for point cloud-based video streaming is expensive and is generally unattainable to an average user due to required setup of multiple depth sensors. We propose a novel hybrid modular artificial neural network (ANN) architecture, which can reconstruct smooth polygonal meshes from a single depth frame, using a priori knowledge. The architecture of neural network consists of separate nodes for recognition of object type and reconstruction thus allowing for easy retraining and extension for new object types. We performed recognition of nine real-world objects using the neural network trained on the *ShapeNetCore* model dataset. The results evaluated quantitatively using the Intersection-over-Union (IoU), Completeness, Correctness and Quality metrics, and qualitative evaluation by visual inspection demonstrate the robustness of the proposed architecture with respect to different viewing angles and illumination conditions.

## 1. Introduction

Reconstruction of three-dimensional (3D) depth-based geometry is one of the core problems in computer vision with commercial applications. These applications range from importing 3D scanned assets into video games and virtual reality (VR) applications [1], gesture recognition [2], indoor mapping [3], recreating environments in movies, recreating evidence and crime scenes in digital forensics [4], designing of dental implants and prosthetics [5], performing Building Information Modelling (BIM) in construction industry [6], environmental perception for industrial/service robots [7], and preserving cultural heritage in museums [8]. However, to this day, the systems that provide 3D scanning capabilities are expensive and are generally unattainable for an average user. Yet, the desirability to have the 3D scene reconstruction is so crucial that the researchers propose new methods that aim to transform RGB images into depth cloud, without the need for additional hardware [9], so that 3D scanning systems would be more affordable and accessible.

We cannot expect the user to either have expensive laser depth scanners which are capable of great accuracy of scanned objects, or an array of sensors which would be capable to pick up all regions occluded by other objects or even self-occlusion and we cannot expect from a general user to be bothered with taking time to precisely scan the entirety of the object so that it would be reconstructed incrementally [10,11] based on delta frames and camera localization. For example, many classical scene reconstruction algorithms rely on simultaneous localization and mapping (SLAM) [12], in order to scan the entirety of 3D objects in the environment, which is then converted either into point-cloud, voxel-cloud volume or triangulated into a mesh. Unfortunately, incremental algorithms tend suffer from one major flaw: changes in scene can create corruptions in the mesh [13]. This makes the application of such approaches unstable in real-world scenes, where objects rather than the view perspective move in space. Other methods such as space carving [14] bypass some of these issues by performing subtractive reconstruction from multiple perspectives with an addition of mask. However, this method assumes that we can accurately extract the mask of an object, which can prove to be very difficult in some aspects due to adverse illumination conditions.

Another approach employed by some of the most successful reconstruction algorithms is to use a priori knowledge of the objects to be reconstructed [15,16,17]. While these methods have shown great recall capabilities and are less prone to errors do to a priori knowledge, they still depend on illumination conditions as the RGB cameras only capture the visible light spectrum, which may cause distortions in case of dim light and would be impossible to use in dark environments.

With the increasing amount of available low-cost consumer-grade depth sensors such as *Microsoft Kinect* [18], *Intel RealSense* [19], and depth cameras becoming a standard feature in some flagship mobile phones, we are moving towards an era where RGB-Depth (RGB-D) sensors are as common as regular RGB cameras. Object detection and segmentation with RGB-D sensors has been widely used in recent years, such as Canonical Correlation Analysis (CCA)-based multi-view Convolutional Neural Networks (CNN) [20], using regular point clouds in addition to multi-views for point cloud recognition [21], fusing CNNs with simultaneous localization and mapping in order to perform object segmentation [22], employing multi-modal deep neural networks and Dempster Shafer evidence theory to achieve the task of object recognition [23], or adopting multifoveated point clouds [24].

Applying data acquired from RGB-D sensors is a logical evolution of the reconstruction algorithms as the non-stereoscopic (two RGB lenses side-by-side simulating binocular vision) depth sensors are less dependant on ambient light conditions and are capable of capturing even in pitch black environments using infrared projectors, albeit are still prone to speckles due to the properties of object surface [25,26]. There have already been attempts to achieve surface prediction by using depth [27] and silhouettes [28] performed experiments mostly consist of synthetic data. On the other hand, these cameras have limitations too. While RGB frames are generally difficult to segment due to different textures and colors [29] and it is generally easier to segment an object from a noisy background using RGB-D sensor from a sufficient distance, objects in close proximity to each other are difficult to be segmented due to their depth values being very similar. Furthermore, commercially used depth sensors tend to suffer from distortions when projecting infrared (IR) laser [30].

We present a hybrid neural network architecture, capable of reconstructing smooth polygonal meshes from a single depth frame, using a priori knowledge and still being capable of running on low-end RGB-D sensor devices in intractable frame rates. The aim is not to scan the 3D geometry, but rather a stream of depth data, which can later be used to recreate 3D holographic objects. The structure of this paper is organized as follows: Section 2 describes the proposed modular neural network, reconstruction algorithm for 3D object recognition, and network training; Section 3 presents experimental results of the proposed network architecture; and finally, Section 4 discusses the results and concludes the article.

## 2. Materials and Methods

### 2.1. Architecture of Hybrid Neural Network

The proposed hybrid network consists of a single classifier network that branches off into n-reconstruction networks (in comparison to standard methods of having a single neural network performing both of these tasks at once). For our hybrid ANN architecture we adopted the hierarchical approach. The ANN consists of a single predictor node and multiple reconstructor nodes, where each reconstructor node is dedicated to recognizing either a specific object or a group of similar objects. This allows more easily training additional types of objects without having to re-train for reconstruction or facing the risk of loosing existing gradients by training on additional models [31]. This adds some modularity to the system while also giving the benefit of reducing the training time due to low iteration count required as the Adam optimizer [32] manages to converge the model in very few iterations generally under 50, depending under model complexity.

**Figure 1:**
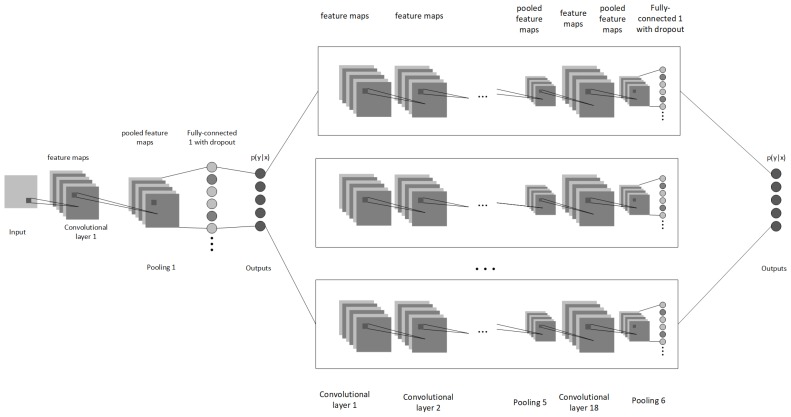
Architecture of hybrid neural network consisting of discriminator and reconstructor. The node accepts depth data as an input, applies a 3 × 3 max-pool convolution layer with a stride of 2 and 32 samples. Following the convolution layer a dropout layer of P(x) = 0.2 is applied to avoid overfitting. The final discriminator layer is the fully connected one-hot layer. The result of the classifier helps us pick the best suited reconstruction neural network for the observed object. Afterwards the same input is sent into appropriate reconstruction neural network which then performs deep convolutions and sends it to fully connected layer which predicts the voxel cloud of the object.

For the discriminator ANN in the hybrid network (Figure 1), we use a simple one hot CNN, which takes an input of 320 × 240 depth frame and runs it through a convolution layer. In convolution layer, we create 32 samples by using a 3 × 3 kernel with max-pooling function, downsampling the original image by a factor of two. After convolution layer we add random noise to the output by using a dropout layer with a chance of P(x)=0.2, which allows for better generalization. Finally, we flatten our output into 1-dimensional tensor and run it through 256 neuron density layer with the output being returned as one-hot encoded array. For all of our layers we used Rectified Linear Units (ReLUs) [33] as they have been shown to give great results in conjunction with CNNs [34]. Finally, we compute the loss using softmax cross-entropy (1) in order to discriminate between different types of object classes, where $y_{0}$ is ground truth value, *p* is predicted value. Once we have the classifier result, we can select the appropriate neural network best fitting for the reconstruction of the observed object frame. Thus the hybridization of these two neural networks allows us to have desired modularity in our method.

(1) $$H\left(y_{0},p\right)=-\sum y_{0}log\left(\frac{e^{p}}{\sum e^{p}}\right)$$

**Figure 2:**
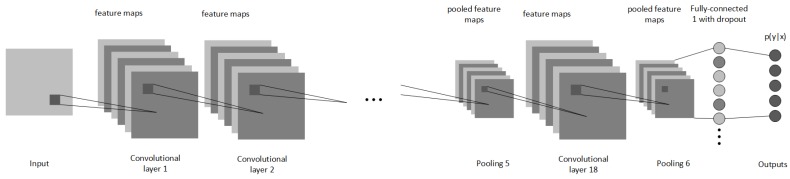
A single branch of ANN used for reconstruction of voxel space.

A single node that is used for reconstructing the voxel volume is shown in Figure 2. The reconstruction ANN adopts the convolutional encoder layers from *PointOutNet* [15] architecture as it has shown to have good encoding capabilities. However, we modified the decoding components. First, we added a dropout layer with P(x)=0.4 for increased generalization, following a 512 neuron density connected layer. The final layer is the 32 × 32 × 32 voxel space layer. While all other layers use ReLU as activation function, the output layer uses the sigmoid function to clamp the output ranges B∈[0;1]. For a loss function, mean square error (Equation (2)) is used, where *p* is prediction, $y_{0}$ is ground truth, *n* is the number of elements in batch. Please note that using the mean value instead of absolute loss creates a better network topology, as the latter may fall into a local minima and constantly generate the same output.

(2) $$H\left(y_{0},p\right)=\sum \frac{{\left(p-y_{0}\right)}^{2}}{n}$$

### 2.2. Reconstruction Algorithm

**Figure 3:**
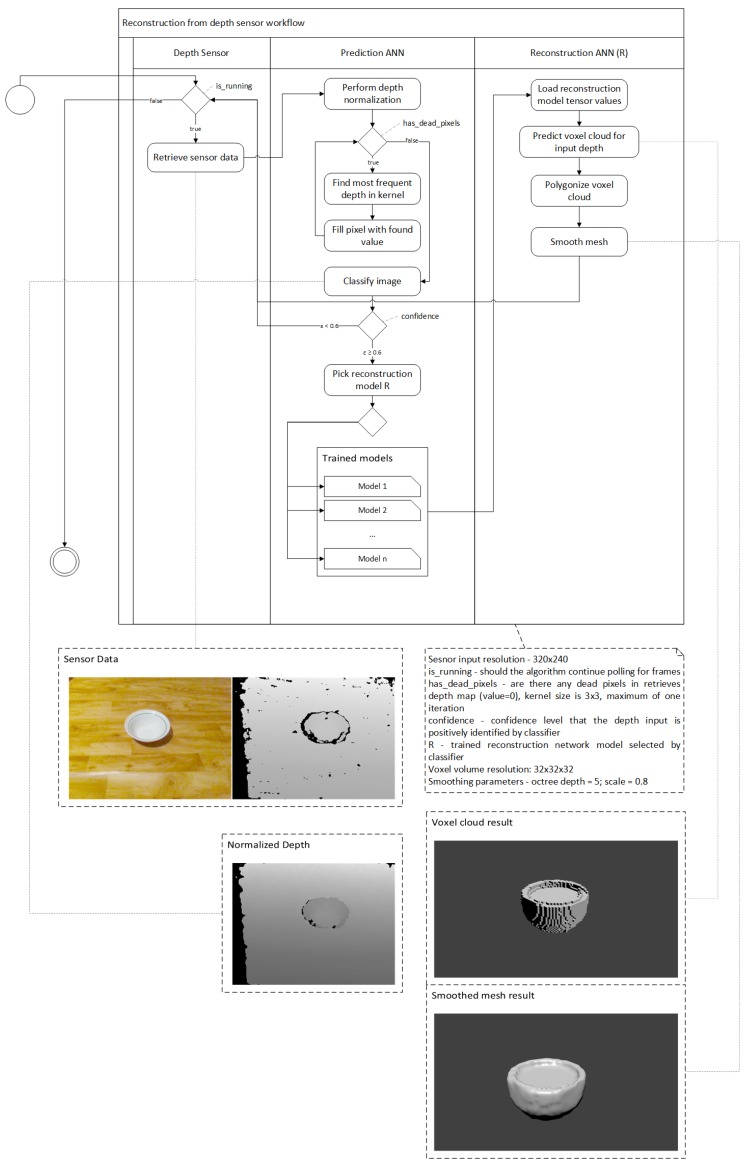
Depth sensor data is captured and sent to classifier ANN. If classifier network recognizes the object, the sensor data is sent to reconstruction ANN, otherwise the frame is dropped. Reconstruction ANN generates the voxel cloud. Voxel cloud is turned into polygonal mesh, and mesh smoothing is applied.

The proposed 3D reconstruction algorithm (Figure 3) consists of three main steps: prediction, reconstruction and post-processing. In the prediction step we use depth sensor data as our input in order to select the reconstruction network, if its was pre-trained. Once the reconstruction ANN is decided the input is then fed to the network to perform voxel cloud prediction.

**Figure 4:**
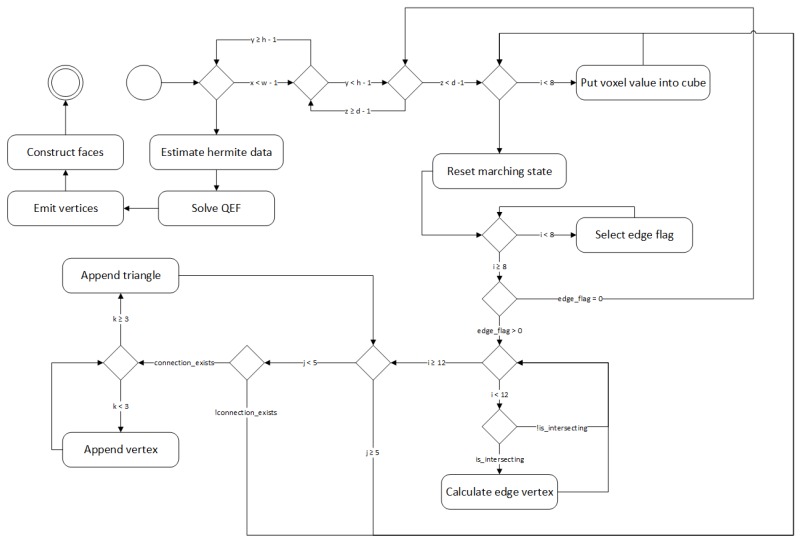
Mesh conversion into polygons via marching cubes.

Finally, the algorithm performs voxel cloud post-processing by turning the reconstruction network output into a polygonal mesh and applying an additional surface smoothing to eliminate noisiness. To use native rendering as provided by graphics pipeline we need to turn the voxel volume cloud into a triangle mesh, which is performed in two steps. First, we convert voxels into triangles via marching cubes [35] using an algorithm presented in Figure 4. We iterate over all voxels in the voxel cloud and create an adjacency cube that is used to determine the shape the voxel should take as follows: we calculate the edges based on adjacency cube. If the adjacency cube ege flag returns 0, we assume that the voxel is inside the mesh and skip it, otherwise we select the edge flag from the marching cube hash table and find the point of intersection of the surface with each edge, if intersections exist we compute the edge vertice positions. Finally, we construct the triangles based on triangle connection table and push them to mesh.

However, this approach produces ambiguities which causes holes to appear in the mesh. Due to the fundamental way non-stereoscopic depth sensors work they are prone to noise. The noise in depth frame acts as *holes* that have a depth value of zero. When using real sensor data, we add additional postprocessing in order to denoise the image as much as possible. We do this by using a kernel method that finds the most frequent value in the kernel and using that as new pixel value (see Equation (3)), where *D* is depth field matrix, *x* and *y* is the coordinates of the pixel on the image.

(3) $$D\left(x,y\right)=\left\{\begin{array}{cc} D(x,y), & \mathrm{if}x\neq 0\\ \hat{D}\left(i,j\right)\;\mathrm{for}\;i=x-2,\ldots ,x+2\;\mathrm{and}\;j=y-2,\ldots ,y+2, & \mathrm{otherwise} \end{array}\right.$$

Furthermore, the generated mesh is somewhat blocky. To mitigate this issue, we further apply smoothing by applying dual contouring [36] on the generated mesh.

### 2.3. Network Training

**Figure 5:**
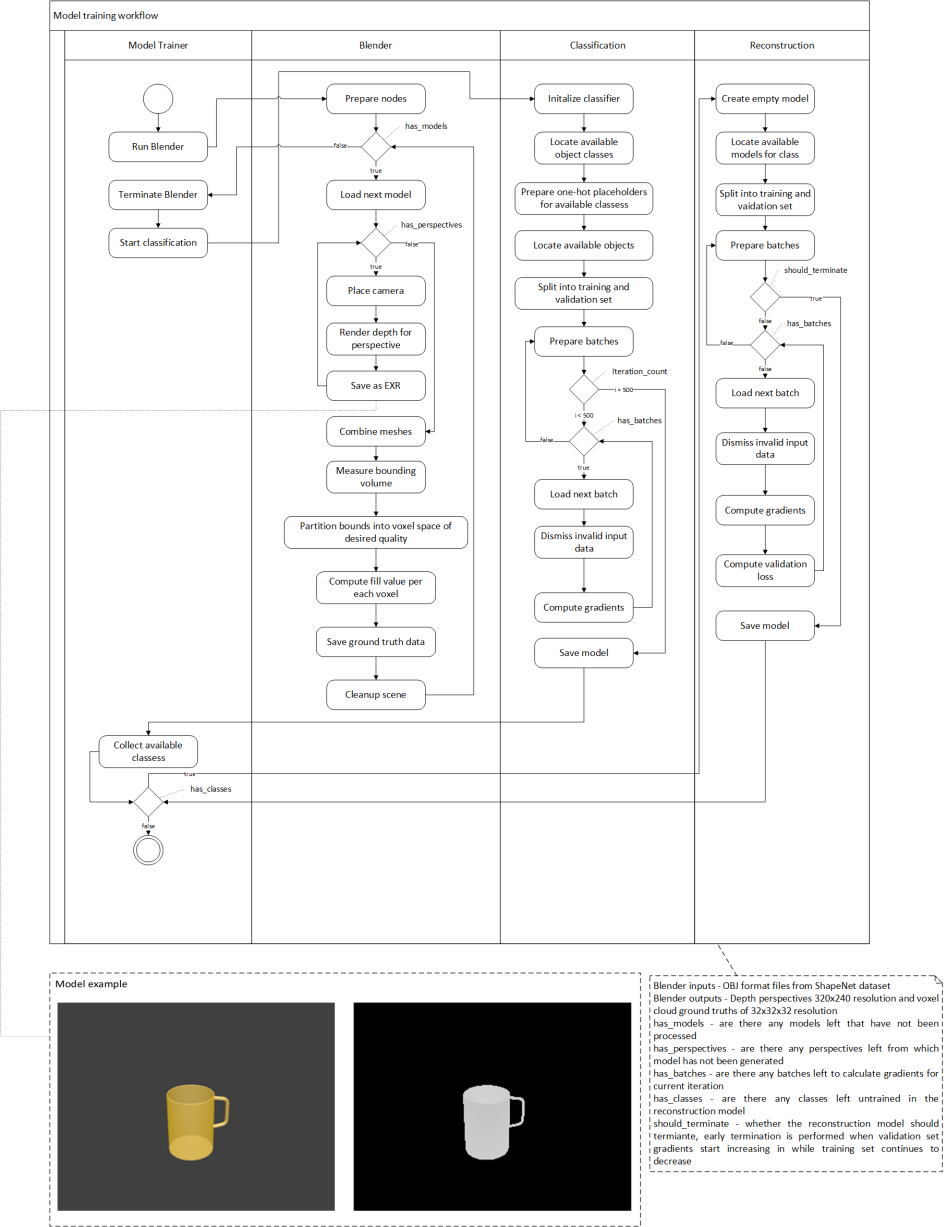
Overview of workflow for *ShapeNetCore* data set preparation and model training.

For neural network training and validation, we use the *ShapeNetCore* dataset and *Blender* in order to generate appropriate depth images and ground truths of voxel cloud. To train the neural network, first, we find all available objects that we are working with and separate them into different objects. Once that step is complete, we pick the first category and load a single *OBJ* object from that category. After the object is loaded, we use *Blender* to render depth fields for each object from different angles. We have selected values the perspectives in such a way that the object would be rendered from all 45° and 90° angles at distances of 1 and 1.5 units, except the bottom, giving us a total of 48 perspectives. We save the perspectives as *OpenEXR* format as unlike standard image formats *OpenEXR* is linear, allowing us to retain all depth range values, which standard non-lossy image formats would loose due to the limitation of 32 bits per pixel [37]. Furthermore, as our network is trained only on depth frames, we do not encounter any problems related emulating lighting as opposed to when choosing Lambert, Phong, PBR, etc. shading models for realistic lightning in RGB enviroments. After we have rendered the given object mesh into depth fields, we perform geometry voxelization as suggested in [38]. This is done by partitioning the geometry boundaries into equal sized cells. The size of the cell is chosen based on the largest object axis. Once the space is partitioned, we iterate over all cells and calculate if the cell should be filled or not, the state is determined using ray-triangle intersection [39]. After the model is processed, we continue with all the models in the class until none are left and move on to next class, we continue this until no classes or objects are left. When data preparation step is complete we perform our training. This is done in multiple stages. First stage consists of training classifier network to recognize the object class so that an appropriate network can be chosen afterwards. Once the classification model has converged or we reach 500 iterations we train each class individually on a new neural network, while saving tensor values for each network. An UML activity diagram in Figure 5 demonstrates this process. Due to automatization of very large quantities of models, automatic depth generation may fail due to irregular object sizes, mainly very thin objects like knives. Therefore, we add an additional check to filter out invalid object inputs such as empty frames and very few clustered pixels that could potentially spoil the training gradients.

### 2.4. Dataset

**Figure 6:**
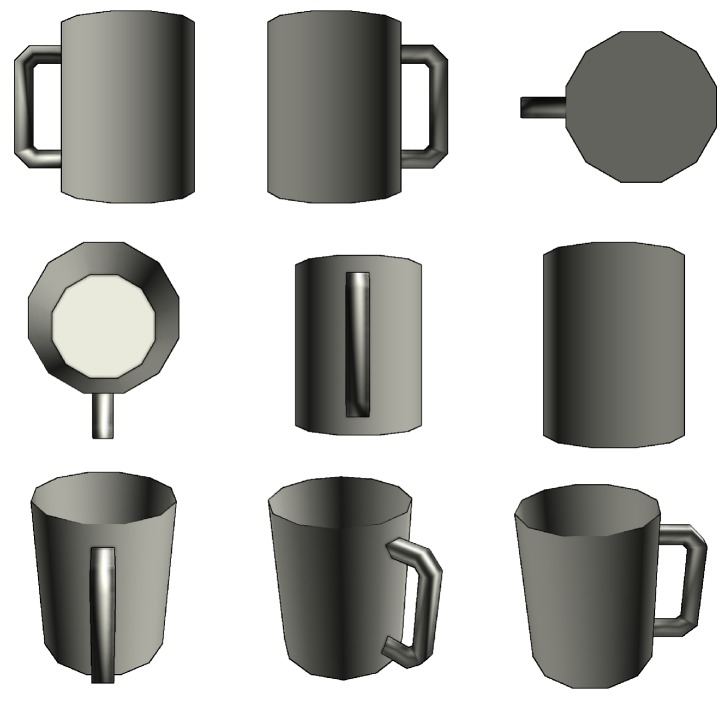
An example of a model from *ShapeNetCore* dataset.

**Figure 7:**
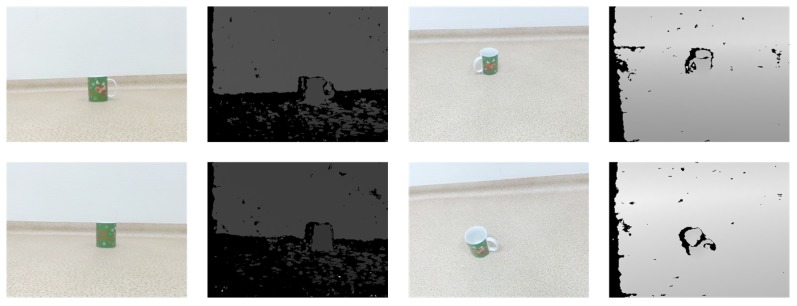
An example of real depth sensor data set captured by *Intel RealSense ZR300* captured from different vantage points.

3D recognition depends on a priori information about desired objects. Therefore, it is a requirement to have a good labeled element dataset. However, for 3D object recognition there are a few such datasets that are more limited. Our main sources are *ShapeNetCore*, a subset of *ShapeNet* [40] dataset that has clean 3D models and manually verified categories, and real-world data captured by the *Intel RealSense ZR300* (Intel Corporation, Santa Clara, CA, USA) device. An example of 3D models provided in *ShapeNetCore* dataset is shown in Figure 6, and an example of real depth sensor data acquired by *Intel RealSense ZR300* is given in Figure 7. While we use *ShapeNetCore* as a source of training data, we also use real depth sensor data for visual validation and testing in real-life applications. This is mainly due to not having ground-truth data for real objects, which unlike virtual model datasets would allow us to extract all the necessary features.

We also have explored different subsets of the *ShapeNet* database. However, these models have proved to be problematic due to their shapes not being properly normalized and aligned as opposed to *ShapeNetCore*, which is undesired effects for training. Therefore, the only model we used from *ShapeNetSem* for our experiments was *Book*, which had the worst recall rates of all models due the problems specified previously.

### 2.5. Evaluation

The goal of reconstruction is usually to achieve a difference between the reconstruction and the ground truth as small as possible. We define reconstruction quality by using *Intersection-over-Union* (IoU) metric [41] as defined by Equation (4), where *A* denotes a turned on voxel in ground truth and *B* denotes a turned on voxel in prediction, and *P* is conditional probability.

(4) $$IoU=\frac{P(B|A)}{P(B|A)+P(\neg B|A)+P(B|\neg A)}$$

We also use the *Completeness*, *Correctness* and *Quality* metrics [42]. *Completeness*, also known as *Producer’s Accuracy* and *Detection Rate*, is the ratio of voxels in ground truth that were reconstructed:

(5) $$Completeness=\frac{P(B|A)}{P(B|A)+P(B|\neg A)}$$

The *Correctness* metric shows how well the reconstructed voxels match the ground truth:

(6) $$Correctness=\frac{P(B|A)}{P(B|A)+P(\neg B|A)}$$

The *Quality* metric gives a combined value that balances both correctness and completeness as follows:

(7) $$Quality=\frac{Completeness\cdot Correctness}{Completeness+Correctness-Completeness\cdot Correctness}$$

## 3. Results

### 3.1. Experimental Settings

The experiments were performed on two different computers: (1) a computer workstation containing *nVidia 1070* graphics card, *Intel i7-4790* processor and installed *16 GB of RAM* which managed to achieve an average of 151 frames per second, and (2) a laptop with a *nVidia 960M* graphics chip, *Intel i5-4210H* processor and installed *12GB of RAM*, which was still able to achieve an average of 28.88 frames per second. We think that both computers represent the range of consumer devices, while the achieved graphics processing speed should be enough for most applications that would use consumer grade depth sensors. Please note that the proposed reconstruction algorithm is GPU bound, therefore we are interested in specifications of the graphics chip.

### 3.2. Quantitative Results

The quantitative results of the proposed algorithm during classification task can be observed in Table 1, as we can see the median recall rate for classification task is close to 84% in the classification task.

The qualitative results for the proposed reconstruction neural network are presented in terms of the IoU metric in Table 2.

**Figure 8:**
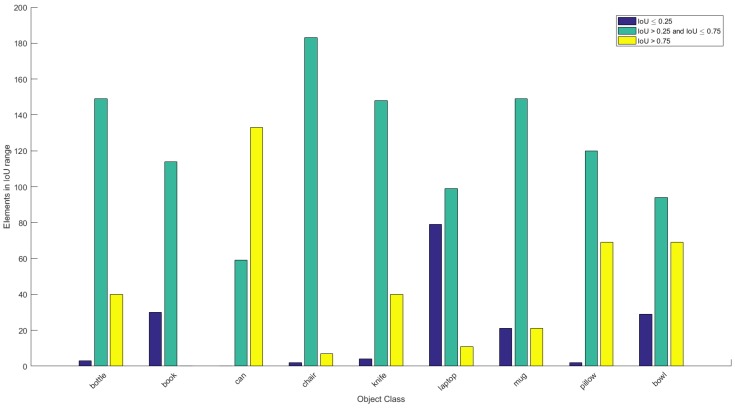
Histogram of the IoU values for heuristic comparison of reconstruction results.

Please note that the IoU metric values presented in Table 2 do not fully capture the quality of the reconstruction due to the low minimum values skewed by failed reconstruction. Therefore, we differentiate the IoU values into three groups of *Poor*, *Good* and *Excellent* quality. We have selected the IoU values corresponding to said groups based on the heuristically set threshold values for the best and worst results when inspecting the models visually. We assume *Poor* quality reconstruction is not able to reach IoU of 0.25, and *Excellent* quality reconstruction has the IoU value exceeding 0.75, while the *Good* quality has IoU∈ (0.25, 0.75]. As we can see from Figure 8, a majority of reconstruction results fall into *Good* category, letting us assert that we achieved the desired goal. However, we still have outliers, such as *Laptop* and *Book*. The poor quality of *Book* reconstruction can be explained by training set being the least diverse of all, which, unlike other sets, has not been properly normalized. Poor reconstruction of *Laptop* may also be caused by poor training set as all of the training models, which contain only opened laptops. To present an overall evaluation of quality, we present the percentage of good and excellent reconstructions with IoU≥0.25 in Table 2.

**Figure 9:**
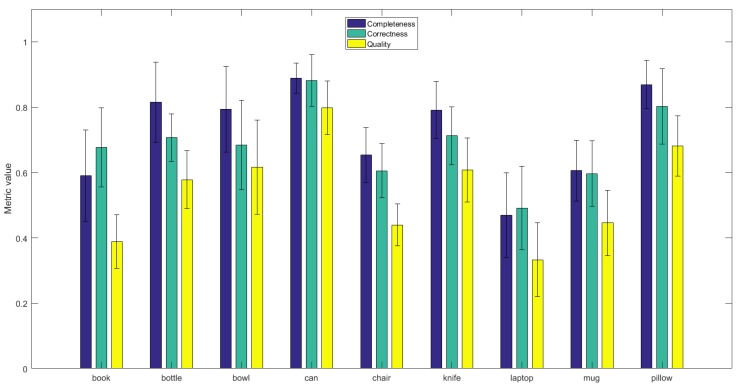
Comparison of reconstruction results using *Completeness*, *Correctness*, and *Quality*) metric values.

The values of *Completeness*, *Corectness*, and *Quality* are summarized in Figure 9. More simple objects with round shape (such as *Can* or *Bottle*) were reconstructed with a larger accuracy.

**Figure 10:**
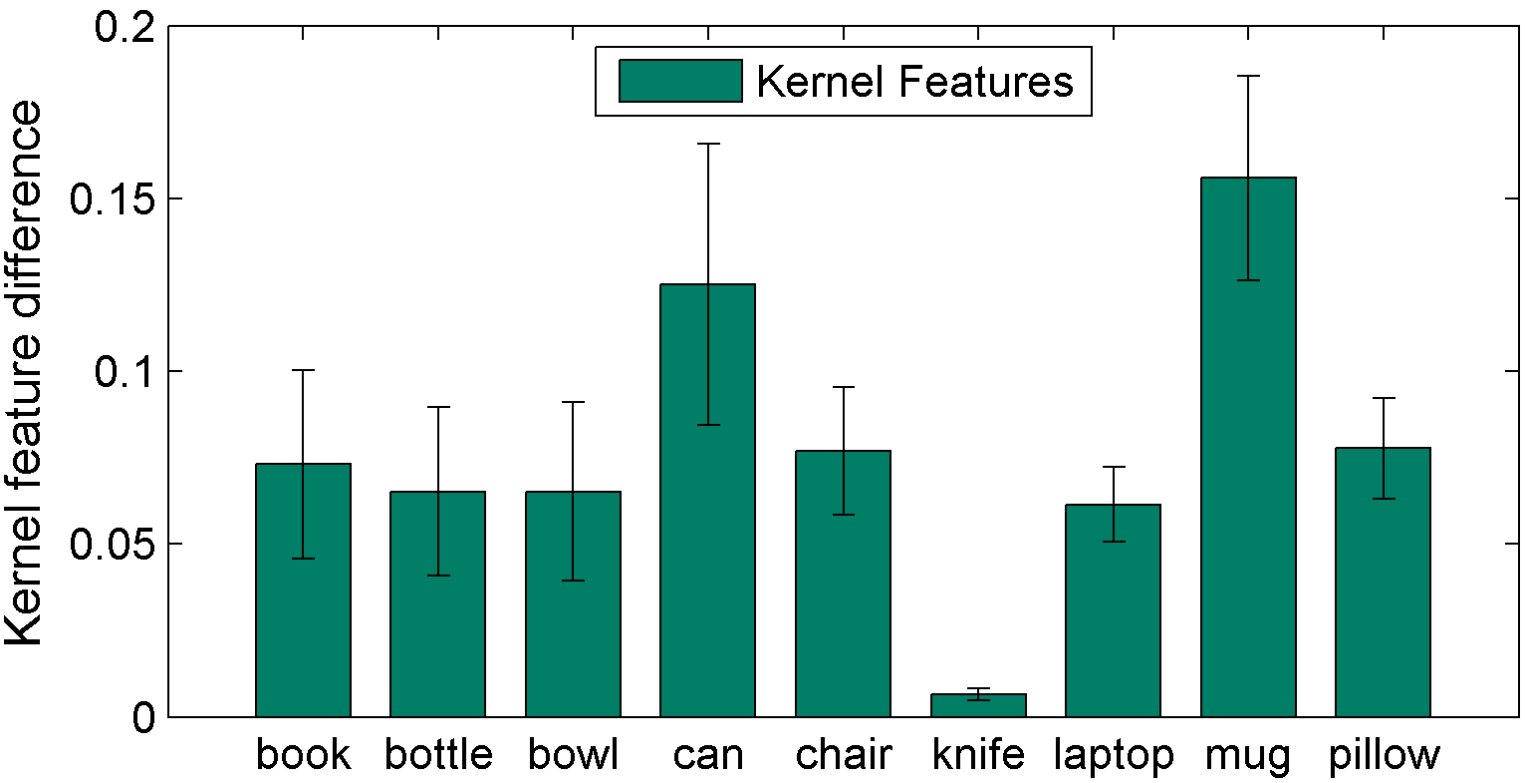
Comparison of kernel features when using 3 × 3 kernel to identify similarities between objects. Zero indicates that kernel features are identical to those of ground truth.

We also have compared object similarity based on its kernel features (see Figure 10). This was done by computing the average a sliding 3 × 3 kernel and comparing to the features found in the ground truth object. Difference between features indicates drift from the expected ground truth, while 0 indicates that features are identical.

### 3.3. Visual Comparison of Reconstruction Results

**Figure 11:**
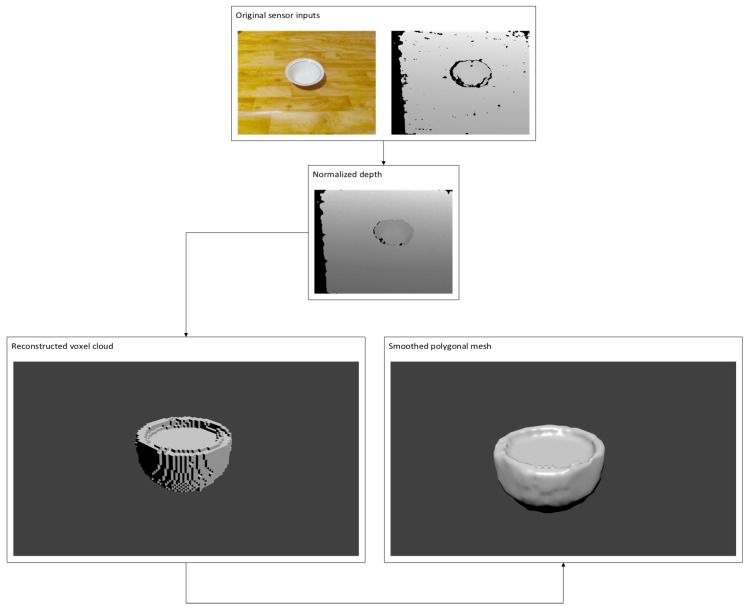
Visual comparison between inputs and outputs.

As we do not have ground truths for real sensor data we can evaluate the results qualitatively by visual inspection. In Figure 11, we provide an example of real depth sensor data and the reconstruction results. For this example, we take an RGB-D frame using a depth sensor used for reconstruction. We use RGB sensor data only as reference point for us to inspect the quality of reconstruction, as the data is not used in the algorithm. We normalize the given depth frame as described in Figure 3, classify it in order to select the correct reconstruction network and forward data to the trained reconstruction model. Once we receive the voxel cloud, we transform it into polygonal mesh and apply smoothing. Finally, we inspect it visually using *Blender*.

**Figure 12:**
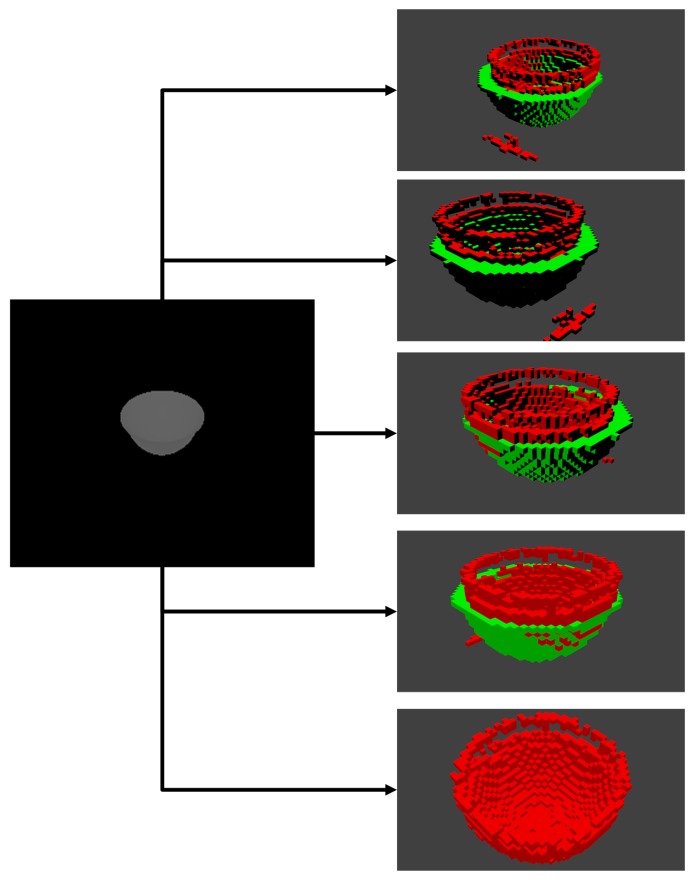
An example of visual comparison ground truth and prediction on the validation set of *Bowl* shape. Green color denotes ground truth, and red color shows prediction.

We also visually compare predictions on synthetic data for our existing models. In Figure 12, we can see the validation results from multiple angles for an example of synthetic depth input of *Bowl* object. The predicted voxel cloud (*red*) value does resemble the object depth field quite well, including the inlet of the bowl, the difference between ground truth (*green*) and prediction are definitely noticeable albeit majority of differences can be considered as negligible. For example, the predicted value is slightly offset from the ground truth center, and predicted bowl is slightly higher then ground truth. However, there are some more important defects, like holes in the predicted voxel cloud and islands of disjointed voxels.

We have collected frames for each of the trained objects in order to inspect reconstruction quality for each class. The results are presented in Table 3 with frames taken at extreme angles, in an attempt to test the reconstruction network. The results show that the proposed neural network architecture is robust against such manipulations and was still able to predict the general shape of an object. The depth frames of *Bowl* captured by *Intel RealSense* have been reconstructed properly in terms of shape, albeit we can see some issues with the inner part of the bowl. In the first reconstruction (see the 1st row of Table 3), the reconstructed bowl is very shallow. In the second reconstruction (see the 2nd row of Table 3), the reconstructed shape has multiple artefacts inside it, although due to the localization of the noise it may be attributed that depth map in question being a lot noisier. The *Book* dataset managed to reconstruct the basic shape of the object. However, it contains an additional appendage which does not seem to be immediately obvious in the depth field. The *Knife* dataset has managed to reconstruct the shape very well, retaining handle-to-blade ratio and seemingly recognizing smaller dents in the handle. Neural network managed to reconstruct the *Bottle* without any obvious glitches. However, we can observe in the RGB image that the object has a narrowing at the top which the network did not manage to capture. However, we still can see that the specific part of object is noisy as well, as *Intel RealSense* was not able to capture it properly. However, due to the 32 × 32 × 32 voxel density we are using, such details would most likely not be visible anyway.

ANN that is trained to reconstruct *Pillow* has managed to perform the task relatively well. However, we can see from the RGB image that the pillow had odd corners, which were not captured by reconstructing ANN. The *Mug* was one of the most complex objects in our training set. However, the reconstructed object is definitely recognizable as a mug. However, we can see that with so many errors in the reconstructed voxel cloud, the smoothing algorithm had trouble while polygonizing the mesh. The *Chair* was reconstructed well and is recognizable as a chair. Unfortunately the full detail of chair’s legs was not captured. The *Notebook* also is easily recognizable, although the polarized glass screen of the notebook appears as black in *Intel RealSense* frame. While this may cause a lot of issues due to network not being trained for it, reconstruction has failed in other places instead. The final dataset consists of *Bottle*, which faced the issue with *Intel RealSense* being unable capturing PET objects, which has caused the depth map to be completely garbled, while the reconstruction results are not recognizable.

**Figure 13:**
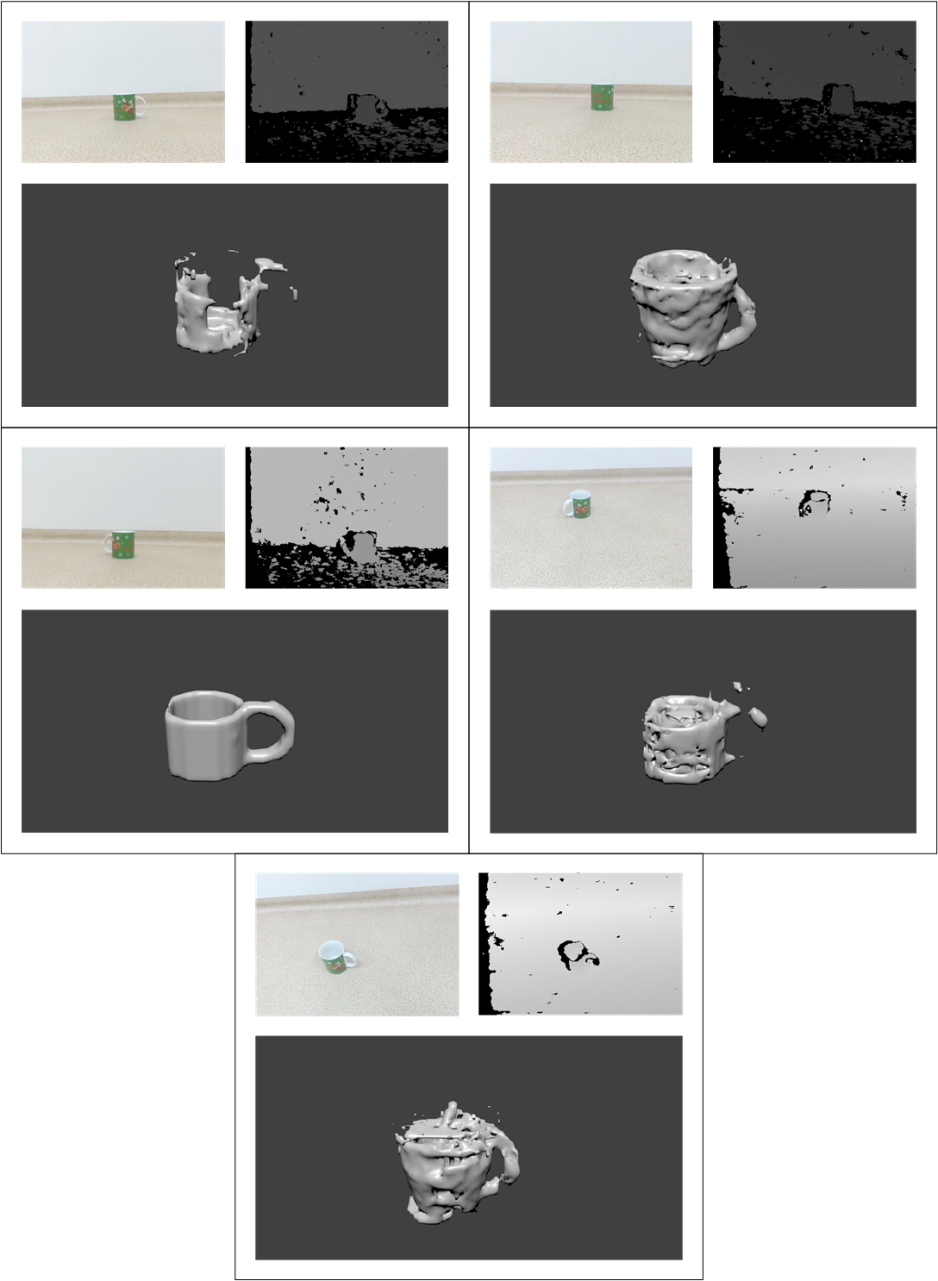
Visual comparison of the same *Mug* object from varying perspectives.

In Figure 13, we present an example of the results of reconstruction for the same object captured from different viewing angles. In one of the images we can see that the handle of a *Mug* is not present in the RGB camera. However, the ANN was still able to infer that the mug should have a handle as the network was only trained on mugs that have handles. Finally, we can see that in the particularly noisy depth fields, the reconstruction quality has dropped significantly, meaning the there was not enough data in the corrupted images for us to reconstruct from a single frame.

In Table 4, we present an example of 3D object reconstruction performed in pitch black (row 1) and low (row 2) illumination conditions. we can see that although in RGB images (column 1) *Mug* is poorly visible, the IR camera captures *Mug* (column 2) fairy well and the result of reconstruction (column 6) is easily recognizable.

## 4. Discussion and Concluding Remarks

### 4.1. Discussion

The advantage of the proposed hybrid neural network (HNN) architecture is that unlike non-hybrid approach, which usually requires to re-train the entire network (off-line) or risk loosing existing gradients (on-line) due to network being skewed towards new data points, the proposed HNN architecture is modular and can easily extend the already trained network by adding additional reconstruction nodes, replace already existing nodes with a better trained model, etc. Although adding additional reconstruction nodes requires to re-train the classifier network, the classifier network still is more light-weight and requires less processing power to train. In addition to this we can have different ANN architectures per node, allowing for a specific reconstruction node to have a more precisely selected reconstruction model. Furthermore, this approach gives us the potential to have variable network complexity contingent upon the complexity of the object we desire to reconstruct, further expanding the applicability of the proposed HNN architecture. Such architecture allowed us to create a system capable of reconstructing polygonal mesh of a self-occluding object by using only a single depth frame on lower-end devices.

We believe that further improvements of network architecture are possible to improve the quantitative performance of 3D recognition. Possible venues of future research may include selecting network architecture that manages to converge well and pruning dead neurons [43]; gradually increasing the complexity of the network until desired reconstruction quality is achieved [44]; using neuro-evolutionary and neuro-genetic algorithms in order to find satisfying network solution [45]; improving learning of networks by using metaheuristic control mechanisms [46]; or using video feed instead of a single frame of an object as multiple depth frames from a single perspective can actually reveal new features [47] thus improving the recall rate, with recurrent neural networks (RNN) being one of the biggest contenders in predicting sequential data [48,49]. Moreover, additional functionality in the method such as solving homography would allow us to extract the transformation matrix of the object, allowing the system to be used for such applications as Virtual Reality in conjunction with Augmented Reality. Finally, using RGB sensor frames in conjunction with depth frames may add some missing features to improve the recall rate even more [50,51].

### 4.2. Threats to Validity

**Figure 14:**
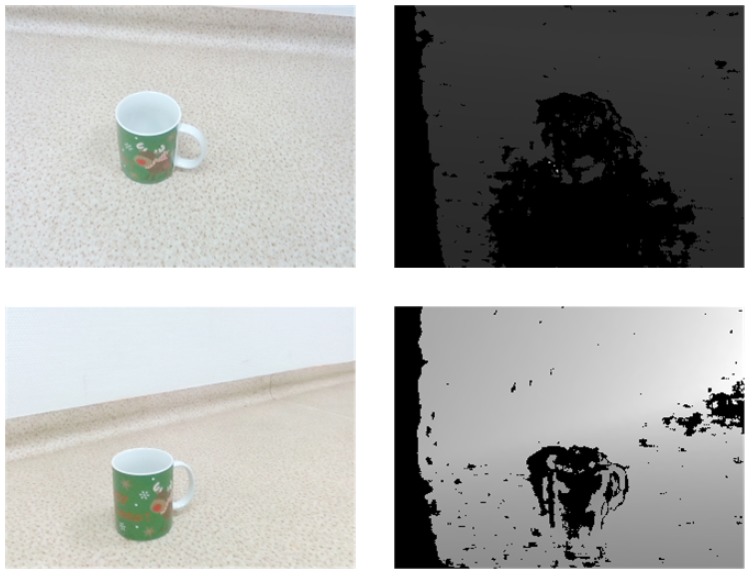
An example of corrupted frames.

A relatively old *Intel RealSense* device was used for the preparation of real-life training dataset which introduced a limitation as the used device did not provide valid depth information if placed too close to the object, while simultaneously being unable to capture the minute details. This has limited us to relatively large (at least 8×10 cm) objects that we can use for reconstruction as putting the camera too close to an object would result in frame corruption (see an example given in Figure 14), while placing the camera far enough (reliable depth capture range is 0.55 to 2.8 m distance) to use the depth sensor would cause the object features to be indistinguishable from the background.

Moreover, the *Intel RealSense* device was unable to properly capture glass surface, e.g., a laptop screen, or translucent PET plastics, which resulted in creating holes and distortions in depth images thus making 3D reconstruction difficult.

### 4.3. Concluding Remarks

We have proposed a hybrid neural network architecture that has managed to reach the goal of reconstructing the shape of 3D objects from different viewing angles using the *Intel RealSense ZR300* device.

The mean IoU value for all objects was in the range of 0.333 to 0.798, obtaining on average 89.5% of good and excellent reconstructions, which is equivalent to the results achieved by other methods, while the reconstructed shapes are easily recognizable by visual inspection.

Furthermore, our proposed architecture allows for an easy extension (requiring very few iterations to train for a new an object type), can work in low illumination environments and has little dependence on ambient lightning, which enables the application of it in more realistic lightning conditions and even where there is no ambient light. This allows our method a broader application spectrum as opposed to other approaches.

## Figures and Tables

**Table 1:** Quantitative results of classifier recall rate on testing set. Our entire dataset was split using 80:20 rule into training and validation sets.

Object	Classification Correctness	Number of Unique Testing Set Objects per Class	Number of Unique Testing Set Objects per Class
Bottle	0.850	399	99
Book	0.754	68	17
Can	0.844	87	21
Chair	0.777	2289	572
Knife	0.898	340	85
Laptop	0.772	460	115
Mug	0.949	172	42
Pillow	0.863	77	20
Bowl	0.843	149	36
Mean	0.839	449	112

**Table 2:** Comparison of qualitative reconstruction comparison between prediction and ground truths using the IoU metric, and the number of different objects in training set.

Object	${\mathrm{IoU}}_{\min}$	$\bar{\mathrm{IoU}}$	${\mathrm{IoU}}_{\max}$	Percentage of Good and Excellent Reconstructions
Bottle	0.220	0.578	0.980	98.438
Book	0.104	0.389	0.707	79.167
Can	0.524	0.798	0.996	100
Chair	0.239	0.440	0.986	98.958
Knife	0.201	0.608	0.944	97.917
Laptop	0.044	0.333	0.984	58.201
Mug	0.149	0.446	0.952	89.005
Pillow	0.229	0.681	0.995	98.953
Bowl	0.106	0.617	0.995	84.896
Mean	0.202	0.543	0.949	89.504

**Table 3:** Visual qualitative reconstruction results. Table shows: RGB frame, infrared (depth) frame, normalized depth frame; reconstructed voxel cloud; polygonized and smoothed voxel cloud; an example of a similar object in training set.

RGB	Depth	Normalized Depth	Voxel Cloud	Mesh	Training Data
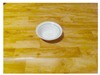	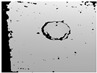	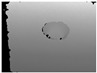	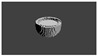	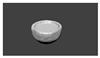	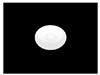
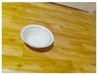	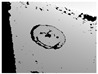	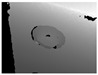	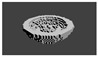	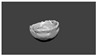	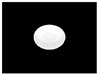
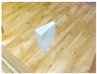	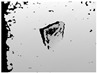	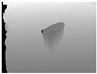	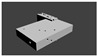	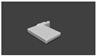	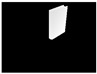
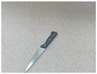	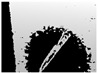	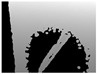	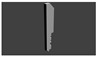	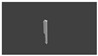	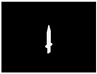
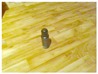	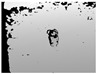	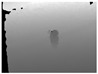	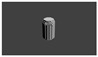	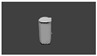	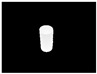
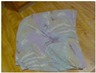	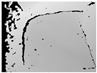	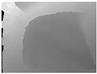	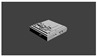	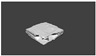	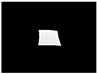
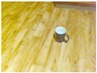	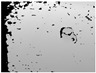	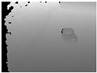	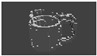	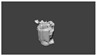	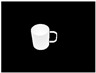
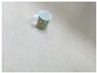	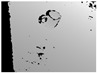	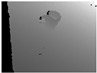	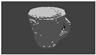	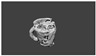	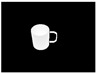
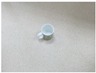	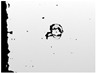	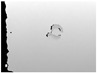	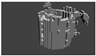	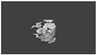	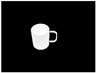
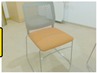	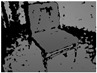	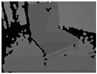	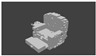	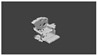	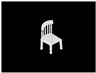
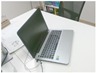	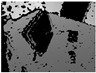	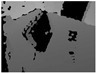	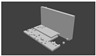	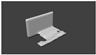	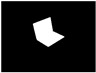
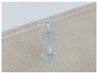	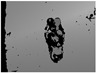	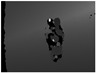	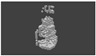	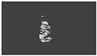	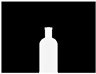

**Table 4:** 3D reconstruction in low illumination conditions: RGB frame, infrared frame, depth frame, normalized depth frame; reconstructed voxel cloud; polygonized and smoothed voxel cloud.

RGB	Infrared	Depth	Normalized Depth	Voxel Cloud	Mesh
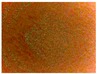	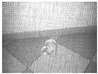	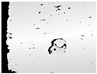	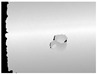	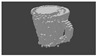	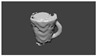
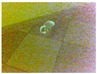	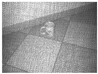	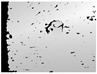	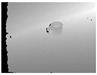	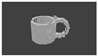	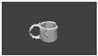
